# Detecting referral and selection bias by the anonymous linkage of practice, hospital and clinic data using Secure and Private Record Linkage (SAPREL): case study from the evaluation of the Improved Access to Psychological Therapy (IAPT) service

**DOI:** 10.1186/1472-6947-11-61

**Published:** 2011-10-13

**Authors:** Simon de Lusignan, Rob Navarro, Tom Chan, Glenys Parry, Kim Dent-Brown, Tony Kendrick

**Affiliations:** 1 Department of Health Care Management and Policy, University of Surrey, Guildford, GU2 7XH, UK; 2 Division of Population Health Sciences and Education, St. George's - University of London, London, SW17 ORE, UK; 3 Sapior Ltd, 8 Cheyne Avenue, London, E18 2DR, UK; 4 Centre for Psychological Services Research, ScHARR, University of Sheffield, Regent Court, Regent Street, Sheffield S1 4DA, UK; 5 Hull York Medical School, University of Hull, Hull HU6 7RX, UK

## Abstract

**Background:**

The evaluation of demonstration sites set up to provide improved access to psychological therapies (IAPT) comprised the study of all people identified as having common mental health problems (CMHP), those referred to the IAPT service, and a sample of attenders studied in-depth. Information technology makes it feasible to link practice, hospital and IAPT clinic data to evaluate the representativeness of these samples. However, researchers do not have permission to browse and link these data without the patients' consent.

**Objective:**

To demonstrate the use of a mixed deterministic-probabilistic method of secure and private record linkage (SAPREL) - to describe selection bias in subjects chosen for in-depth evaluation.

**Method:**

We extracted, pseudonymised and used fuzzy logic to link multiple health records without the researcher knowing the patient's identity. The method can be characterised as a three party protocol mainly using deterministic algorithms with dynamic linking strategies; though incorporating some elements of probabilistic linkage. Within the data providers' safe haven we extracted: Demographic data, hospital utilisation and IAPT clinic data; converted post code to index of multiple deprivation (IMD); and identified people with CMHP. We contrasted the age, gender, ethnicity and IMD for the in-depth evaluation sample with people referred to IAPT, use hospital services, and the population as a whole.

**Results:**

The in IAPT-in-depth group had a mean age of 43.1 years; CI: 41.0 - 45.2 (n = 166); the IAPT-referred 40.2 years; CI: 39.4 - 40.9 (n = 1118); and those with CMHP 43.6 years SEM 0.15. (n = 12210). Whilst around 67% of those with a CMHP were women, compared to 70% of those referred to IAPT, and 75% of those subject to in-depth evaluation (Chi square *p* < 0.001). The mean IMD score for the in-depth evaluation group was 36.6; CI: 34.2 - 38.9; (n = 166); of those referred to IAPT 38.7; CI: 37.9 - 39.6; (n = 1117); and of people with CMHP 37.6; CI 37.3-37.9; (n = 12143).

**Conclusions:**

The sample studied in-depth were older, more likely female, and less deprived than people with CMHP, and fewer had recorded ethnic minority status. Anonymous linkage using SAPREL provides insight into the representativeness of a study population and possible adjustment for selection bias.

## Background

Selection bias may distort the results about the effectiveness of a new service [1]. In the NHS nearly all the population are registered with a single family practitioner; and have a single unique identifier (NHS number) which can be linked to health services utilisation, making it possible in theory to quantify selection bias and if needed adjust for it [2]. Although computerised records make it technically straightforward to link population, practice, hospital and clinic data, it is not possible to extract a patient's records without their consent. For a large population based study this is not feasible; and obtaining this consent may result in further bias [3]. Methods are needed which allow selective mining of key variables from individual patients' records to enable researchers to know the extent of any selection bias. Such methods should allow anonymous extraction and linkage of data with only the data needed to make comparisons extracted; and the privacy of the patient is primarily maintained through the researcher not having access to any strong identifiers (e.g. name, date of birth etc.) [4-7].

The Improving Access to Psychological Therapies (IAPT) programme is a Department of Health (DH) quality improvement initiative [8]. The DH also commissioned a comprehensive evaluation of the IAPT programme, which included a case-control study of those referred to the IAPT clinics against age-sex practice matched controls, and an in-depth study of a cohort of people who attended the IAPT clinic and consented to provide further information for the evaluation [9]. IAPT offers a series of stepped interventions, including the use of cognitive behavioural therapy (CBT) which aims to reduce the economic burden to society of psychological illness and enable people to cope better with their mental health problems. The target population for the IAPT programme is people with common mental health problems (CMHP) in primary care, specifically people suffering from depression and/or anxiety disorders. The thresholds of severity of CMHP for referral to IAPT were not always strictly adhered to by referrers. Access to IAPT is further complicated as patients also have direct access to the service without seeing their GP. We linked practice, hospital and IAPT clinic data to conduct this evaluation - linking anonymised data using privacy enhanced fuzzy matching to maximise join quality. We called this process SAPREL - **s**ecure **a**nd **p**rivate **re**cord **l**inkage. The resulting merged data table enabled the tracking of health utilisation of individuals across primary care, hospital services and within the IAPT clinics.

The purpose of this paper is to explore any selection bias in the people referred to IAPT and those who underwent in-depth evaluation. We compares the characteristics of the populations linked using the SAPREL process: (1) The practice registered population; (2) Those referred to IAPT; (3) Uses of hospital services; (4) People with CMHP (a subset within the practice population); and (5) In-depth evaluation group (an enhanced subset within the IAPT population). We contrasted the age, gender, ethnicity and level of deprivation of these five populations.

## Method

### Data sources

Twenty practices consented to participate in this study, 10 each in two localities which piloted the first IAPT services in England. One was within London in an area with a diverse ethnic population; the other a northern city with a predominantly white population. We extracted data from their electronic patient record (EPR) systems using MIQUEST (Morbidity Information Query and Export Syntax) - a Department of Health sponsored application which allows the same data extraction query to be run on different branded EPR systems. These data were extracted, processed and cleaned using well established methods [10,11].

The hospital and IAPT clinic data for these 20 practices were exported using their standard data export methods. Hospital episode statistics, or SUS data (Secondary Uses Services) between 01/10/2007 and 30/04/2009 were retrieved by the information services of the primary care trust of the 2 study sites. Customised output from the IAPT clinic of all those referred between 1/10/2007 and 30/09/08 (on an 'intention to treat' basis) was exported from two different bespoke applications developed specifically to support IAPT clinics. These data were de-identified within the premises where the data were held or accessed, and then subsequently linked using the SAPREL method. This method means that no person identifiable data left the premises where such data were held, and at no time did the researchers hold strong identifiers.

### The secure and private record linkage (SAPREL) process

SAPREL fuzzy linking can be characterised as a deterministic algorithm with dynamic linking strategies determined by the lowest measured link error estimates; (i.e. we flexibly apply the algorithm which appear to generate fewest errors.) The method also incorporates some probabilistic elements to enable wider record matching. The privacy preserving nature of SAPREL flows from its ability to operate on data fully de-identified within each contributing site.

The SAPREL privacy enhanced data linking technology is a "Three-Party Protocol" [12] consisting of the following steps:

1. Normalise and cleanse fields used for linking (dates to ISO format, postcodes to a standard form,)

2 Generate functions of the forename and surname to overcome minor spelling problems (Soundex and Metaphone of various substrings) [13,14]

3. Create derived values from fields used for linking (year of birth from date of birth, deprivation Index from postcodes, and so on)

4. We encrypt every field with a key held at the contributing facility. Fields used for cross-site linking are encrypted again with a common key and a salt (random pad) to prevent dictionary attacks by the data intermediary.

We performed this process at each site contributing data. Where there were no NHS numbers in common the linking process becomes "fuzzy" using the following steps:

1. Generate multiple link strategies (forename, surname, date of birth (DoB), postcode) vs. (surname, DoB, postcode) vs. etc.

2. Estimate the false positives in each strategy via the "Duplicate Method" [15]

3. Choose the link strategy from those with the lowest false positive error estimate that also have the highest number of distinct links. This helps to eliminate strategies with high false negative counts.

### The data set

The GP practice data set included: personal identifier for data linkage - forename, surname, date of birth, NHS number and postcode; demographic information: gender, ethnicity, registered date; and postcode to map to the Index of Multiple Deprivation (IMD) [16] using Geographical Information System (GIS) methods. IMD is divided into deciles of equal sizes, where the first decile (IMD ≤ 5.63) is the least deprived and decile ten (IMD ≥ 45.33) the most deprived. The ethnicity codes are mapped to the National Statistics "5+1" categories [17]. The categories are: Not stated, white, mixed, Asian or Asian British, Black or Black British, Chinese or other ethnic group. We extracted clinical information which enabled us to report whether a patient had a CMHP, namely a diagnosis of depression or anxiety, coded in the clinical computer system. Where we compare the people with CMHP with the other groups we make the comparison with the adult (≥16 years) population. Additional data were extracted for the DH commissioned evaluation, but are not reported in this paper.

We used NHS number (a unique personal ID) to link primary and secondary care data; NHS number was pseudonymised and encrypted and not directly browsed by the researcher. There were a large number of people with a temporary, duplicate and missing NHS numbers (table 1), as well as errors with missing name or gender (n = 609); though this is less than 0.5% of records (n = 152,363). Where a unique NHS number was not available in the primary or secondary care data, that record was discarded. Missing post codes (n = 129) are less surprising as additional post codes are created with building developments. Although for the DH evaluation we separated types of attendance, for this paper we used a single variable to encompass all usage of hospital services, which included out-patient clinics, accident departments, day treatment units and in-patient episodes.

**Table 1:** Missing, temporary or duplicate unique identifiers (NHS number) by practice

Practice number	List size	Duplicate or default NHS number	Missing NHS Number	Total of NHS Number problems	Missing Forename or Surname	Missing Post-code	Demographic data errors	TOTAL ERRORS	Errors as % List size
	N	n	n	**n**	%	n	n	**n**	%

2	13552	0	145	**145**	1.07	2	26	**173**	1.28
14	7190	68	0	**68**	0.95	0	1	**69**	0.96
20	10354	61	0	**61**	0.59	0	0	**61**	0.59
15	5189	49	0	**49**	0.94	0	5	**54**	1.04
11	7611	52	0	**52**	0.68	0	0	**52**	0.68
13	11828	45	0	**45**	0.38	0	2	**47**	0.4
5	6875	6	1	**7**	0.1	0	38	**45**	0.65
19	4715	38	0	**38**	0.81	0	4	**42**	0.89
4	8951	36	0	**36**	0.4	0	0	**36**	0.4
12	5971	31	0	**31**	0.52	1	3	**35**	0.59
1	6661	2	0	**2**	0.03	0	28	**30**	0.45
17	7338	23	0	**23**	0.31	0	2	**25**	0.34
7	9508	0	14	**14**	0.15	0	10	**24**	0.25
6	7951	4	1	**5**	0.06	0	8	**13**	0.16
18	3740	11	0	**11**	0.29	0	2	**13**	0.35
9	4466	4	7	**11**	0.25	0	0	**11**	0.25
16	14708	6	0	**6**	0.04	0	0	**6**	0.04
3	10049	0	2	**2**	0.02	0	0	**2**	0.02
8	2092	0	0	**0**	0	0	0	**0**	0
10	3614	0	0	**0**	0	0	0	**0**	0

**Total**	**152363**	**436**	**170**	**606**	**0.4**	**3**	**129**	**738**	**0.48**

As the NHS number field is poorly populated in the IAPT data, the GP practice and IAPT clinic data only had forename, surname, date of birth and postcode in common. As part of the SAPREL process, various functions of these fields were paired in various combinations. The link strategy with the lowest estimated false positive and false negative count was ultimately selected. Where an IAPT forename, surname, date of birth or postcode was missing - that record was discarded (n = 98; 91 missing postcodes, 7 missing dates of birth).

### Validity of the data linkage

When NHS number is present, and used to link hospital and practice data there is a relatively low risk of failure in the linkage. However, for the clinic data linkage where NHS number is less available we compared the distribution of two readily available variables age and IMD for the patients with linked data with data available for other cases referred to IAPT but not part of the study.

### Statistical methods

We used descriptive statistics to compare the samples. We quoted 95% confidence intervals (CI) and standard error of the mean (SEM) to allow comparison between age groups; and used a t-test to give the probability that age were significantly different. We used Chi square to compare proportions of categorical variables. We used the Wilcoxon non-parametric test to compare the distribution of 5-year age-bands between different populations.

### Ethical considerations

A national research ethics committee (reference No: 08/H0715/101) provided ethical review and the research office of the local healthcare organizations involved provided local site approval. Specific approval was obtained from the former Patient Information Advisory Group (PIAG) for Section 60 exemption for the transient holding of patient identifiable information while it is pseudonymised and encrypted on health service premises (Reference number: PIAG 6-06(h)/2008). The SAPREL process complies with both research and information governance frameworks in England in the use and protection of patient information; and was commended by PIAG as an example of best practice.

## Results

### Age and gender

**Figure 1:**
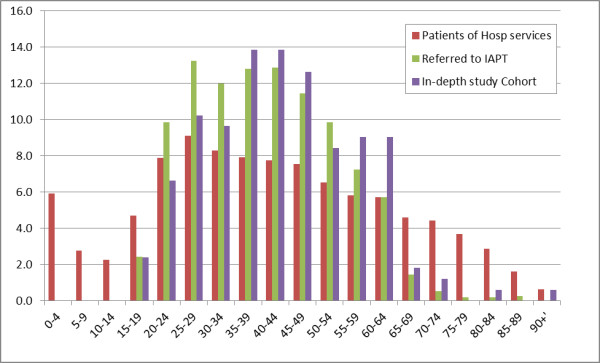
**Comparing the 5-year age bands of people in the in-depth evaluation, referred to IAPT and who use hospital services**.

The in-depth study cohort was roughly three years older than the people referred to IAPT (One sample t-test *p* < 000) and about 0.5 years younger than people with CMHP (One sample t-test *p *= 0.001). The mean for the in-depth cohort was 43.1; CI: 41.0 - 45.2 (n = 166) for people referred to IAPT: mean = 40.2; CI: 39.4 - 40.9 (n = 1118); and for people with CMHP 43.6 years; CI: 43.3 - 43.9 (n = 12210). The practice population was younger than those referred to IAPT (mean age 35.3 years; CI: 35.1 - 35.4 (n = 152302)) though the population attending hospital were a similar age (41.6; Std D = 22.2; CI: 41.4 - 41.8 (n = 60143)). Similarly the age-sex profiles of the practices were not significantly different from the locality populations they were drawn from; Wilcoxon non-parametric test p = 0.133, though there was a slight excess of people between 25 and 34 years old. However, although the people who use hospital services and IAPT clients have similar mean ages, this the majority of people referred to IAPT are in the age bands between 20 and 54; reflecting how initially this service was targeted at people of working age. Nobody below the age of 16 and few people aged 65 and over were referred to IAPT. The in-depth evaluation group was over-represented in the over 35 years age group; and increased use of hospital starts after age 45 (Figure 1).

Women represented just under half of the population; yet female gender is associated with greater use of hospital, common mental health problems, referral to IAPT, and with three-quarters of the in-depth study cohort being female. The gender distributions in the five comparator populations are shown in table 2, with all the differences statistically significant. The proportion of women referred to IAPT is similar to the greater proportion with CMHP, but there is a step up in proportion in the in-depth study cohort. The people referred to IAPT consisted almost entirely of adults of working age (15 - 64), two thirds of whom are women.

**Table 2:** Comparison by gender of practice list, using hospital services, common mental health problems (CMHP), referred to IAPT and part of the in-depth evaluation cohort

	In-depth study cohort	IAPT referrals	CMHP	Use hospital services	Practice list
Female	123	734	8229	33785	75523
	74.10%	65.70%	67.40%	56.00%	49.60%
	
Male	43	384	3981	26457	76779

	25.90%	34.30%	32.60%	44.00%	50.40%

**Total**	**166**	**1118**	**12210**	**60143**	**152302**
	**100%**	**100%**	**100%**	**100%**	**100%**

	Pearson X^2^	Pearson X^2^	Pearson X^2^	Pearson X^2^	NPar X^2^
	*p*< 0.001	*p *= 0.023	*p*< 0.001	*p*< 0.001	*p *= 0.016

### Ethnicity

The level of ethnicity recording in the in-depth evaluation group was about half that recorded for the rest of the population (table 3). Around two-thirds (64.8%) of the population referred to IAPT and over half (53.1%) of the subgroup with CMHP had their ethnicity recorded; while generally ethnicity was recorded for 60% of the practice population and those referred to hospital. The level of recording of ethnicity codes varied significantly between the two demonstration sites (table not shown); the London site had a higher level of recorded ethnicity (81.4%) compared with the northern site (35.1%).

**Table 3:** Rates of recording of ethnicity by patient group

	In-depth study cohort		CMHP		IAPT clinic referral		Use hospital services		GP list population	
No ethnicity code/not stated	113	68.1%	394	35.2%	5732	46.9%	23205	38.6%	62389	41.0%
White	48	28.9%	536	47.9%	4940	40.5%	21318	35.4%	49127	32.3%
Mixed	0	0.0%	10	0.9%	78	0.6%	605	1.0%	1432	0.9%
Asian or Asian British	5	3.0%	91	8.1%	710	5.8%	7470	12.4%	19550	12.8%
Black or Black British	0	0.0%	78	7.0%	639	5.2%	6345	10.5%	15957	10.5%
Chinese/other ethnic group	0	0.0%	9	0.8%	111	0.9%	1200	2.0%	3847	2.5%

**Total**	**166**	**100%**	**###**	**100%**	**12210**	**100%**	**60143**	**100%**	**152302**	**100%**

Over two-thirds of the in-depth study cohort do not have ethnicity recorded in their GP record

**Figure 2:**
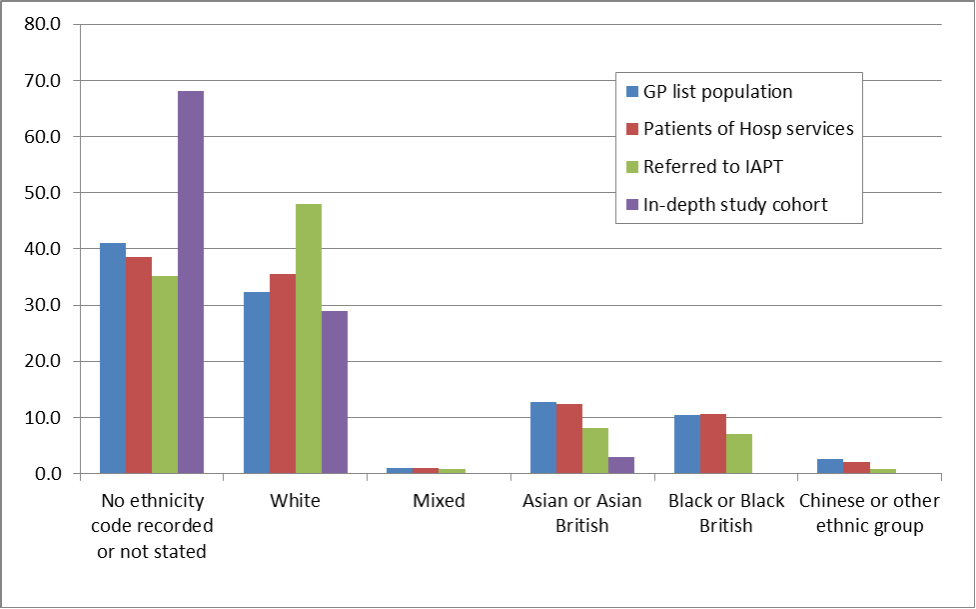
**Proportion of each ethnic group in four study populations (In-depth evaluation, Referred to IAPT, Use hospital services, Registered with a study practice)**.

Around 60% of people in the different study populations have their ethnicity recorded; except in the in-depth study cohort where it fell to just over 30%. White ethnicity is recorded in 32.3%, of the GP practices' population, in 40.5% of those with CMHPs and 47.9% of people referred to IAPT clinics. However, only 28.9% of people in the in-depth study cohort were recorded as having white ethnicity; though this represents 90% of the ethnic recording in this group. There were no members of the in-depth evaluation group with recorded Mixed, Chinese or Black ethnicity; Asian, black and Chinese ethnicity recording was under-represented in the group referred to IAPT (Figure 2).

### Index of multiple deprivation (IMD)

Over 99% of people were mapped to an appropriate IMD decile; less than a thousand out of the 152302 patients (about 0.5%) could not be mapped due to invalid postcodes. The mean IMD scores for the four populations are similar. The mean IMD score for the sample selected for the in-depth IAPT study is 36.6. The equivalent data for those referred to IAPT, people with CMHP, those who use hospital services, and for the population were: 38.7, 37.6, 38.1 and 38.0 respectively (Table 4).

**Table 4:** Mean deprivation (IMD) score for each study population

	In-depth study cohort	IAPT clinic referral	CMHP	Use hospital services	GP list population
**n**	166	1,117	12,143	59,903	151,556
**Mean IMD score**	36.6	38.7	37.6	38.1	38

**95% Confidence Intervals**	34.2-38.9	37.9-39.6	37.3-37.9	38.0-38.2	38.0-38.1

**Figure 3:**
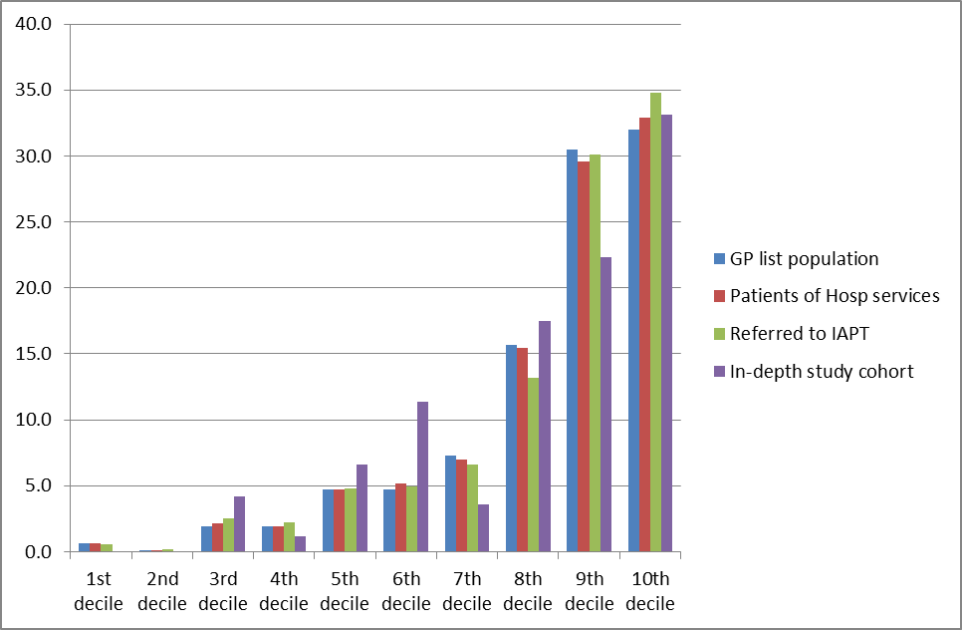
**Distribution of study populations by index of multiple deprivation (IMD) decile**.

The majority (30.5%+32% = 62.5%) of the patients of the GP registered population are in the most deprived 20% (i.e. 9^th ^and the 10^th ^decile). The hospital, CMHP, and the IAPT clinic populations broadly reflected the IMD scores of the GP list population from which they were drawn, with perhaps a small rise in the 10^th ^decile and a small reduction in the 8^th ^decile for IAPT clients compared with the GP list population. The in-depth IAPT study cohort were slightly over-represented (compared with the other three study populations) in the 3^rd^, 5^th ^and the 6^th ^decile (Figure 3).

### People with common mental health problems (CMHP)

The majority of those referred to IAPT were people with a recorded diagnosis of CMHP. However, not everyone referred to IAPT was in the CMHP population, for example, those accessed IAPT directly without a GP referral, and those with mild CMHP without a formal diagnosis in primary care. We therefore compared those with and without CMHP in each of our four groups: in-depth evaluation, referred to IAPT, received hospitalisation, and for the registered population as a whole (Table 5).

**Table 5:** Comparing those with and without common mental health problems within the four study populations

	In-depth study population	Referred to IAPT	Hospital adult population	GP listed adult population
**People with NO record of Common Mental Health Problem (CMHP):**				

% of population	10.80%	30.80%	85.20%	89.90%
Age (SEM)	49.3 (3.4)	40.2 (0.74)	46.5 (0.05)	42.3 (0.05)
Gender (F%: M%)	61.1%: 38.9%	55.8%: 44.2%	55.0%: 45.0%	47.7%: 53.3%
IMD (SEM)	36.8% (3.2)	40.4 (0.72)	37.6 (0.07)	37.7 (0.04)

**People with a Common Mental Health Problem (CMHP):**				

% of population	89.20%	69.20%	14.80%	10.10%
Age (SEM)	42.4 (1.1)	40.2 (0.46)	44.7 (0.19)	43.6 (0.15)
Gender (F%: M%)	75.7%: 24.3%	70.0%: 30.0%	69.7%: 30.5%	67.4%: 32.6%
IMD (SEM)	35.5 (1.3)	38.0 (0.53)	38.1 (0.17)	37.6 (0.13)

**Total population size and differences between people with and without a CMHP**				

Total population size	100%	100%	100%	100%
	(n = 166)	(n = 1,118)	(n = 53,318)	(n = 121,199)
Age difference	-6.9 years	None	-1.8 years	+ 1.5 years
(Significance t-test)	(p < 0.05)	(n.s.)	(p < 0.001)	(p < 0.001)
Gender differences	14.60%	14.20%	14.70%	19.70%
(Pearson Chi-square)	(p < 0.001)	(p < 0.001)	(p < 0.001)	(p < 0.001)
IMD difference	-1.3	-2.4	0.5	-0.1
(Significance t-test)	(n.s)	(p < 0.05)	(p < 0.01)	(n.s.)

In the in-depth cohort those with CMHP were around seven years younger and more likely to be female compared with those with no CMHP; there are no significant differences in multiple deprivation scores. In the group referred to IAPT those with CMHP came from slightly less deprived areas, and more likely to be female compared with those with no CMHP; there were no significant difference in age. In the adult population who had attended hospital the people with CMHP were approximately two years younger, came from slightly more deprived areas, and more likely to be female. Taking the practice population as a whole people with CMHP tended to be a little older and more likely to be female.

### Inter-practice and inter-locality variation

All the practices except one referred patients to IAPT; and two other practices were low referrers. All the practices referred between 0.5% and 2.5% of their adult registered list, which is also between 3.5% and 12.5% of the number of people with CMHP (though not all those referred came from this group). The age distributions in the two localities was not significantly different from the population in the sample practices (Wilcoxon non-parametric tests comparing 5-year age bands) p = 0.58 and p = 0.145 for the northern and southern localities respectively. The northern was almost a perfect match, there was an excess of young adults in the southern locality.

### Validity of the linkage

We compared those referred to IAPT in the study (n = 1,118) with other cases where data were not linked (n = 4,353) and found that they were of similar age: mean 40.2 years, vs. 39.2 years respectively; and for IMD mean score 38.7 vs. 35.5. The distributions of these data were similar between the two groups (See Additional file 1). The age distribution was left-skewed with almost no referrals below age 20 years (as shown for the IAPT referred population Figure 1; Chi-square test p = 0.03). The distribution of IMD showed a similar ranking in both groups with increasing proportions as deprivation worsens (as shown for IAPT referred population in Figure 3; Wilcoxon ranking test suggest no statistically significant difference p = 0.460).

## Discussion

### Principal findings

By linking three differently structured health databases we were able to characterise the population referred and the group who were part of the in-depth evaluation; without knowing the researchers knowing the identities of the people they were linking. The process was able to take place as a result of visits to the practice, hospital or clinic safe haven; and so was mobile and flexible. Had we applied this process *a priori *we would have been able to more purposively sample; applied post hoc it at least allows allowance to be made for population differences.

The practice populations were not significantly different from the locality from which they were drawn. The people referred to IAPT were not exclusively drawn from those recorded as having CMHP; and the group studies in-depth for the service evaluation were relatively older, more likely to be women, and included fewer with a recorded ethnic minority status.

This approach has allowed databases designed to serve a different purposes and using different coding systems to be linked. The SAPREL process also meets with stringent research and information governance requirements for the ethical use and protection of patient information.

The finding also shows that over 60% of the patients in the study practices' populations lived in the 20% most deprived areas as measured by the Index of Multiple Deprivation. However, men, older people and some ethnic groups were apparently less likely to be referred.

### Implications of the findings

These data were linked so that we could conduct a case controlled, before and after study of hospital utilisation of people referred to IAPT, and by the time that permissions were obtained and this linkage completed, the IAPT evaluation programme was well underway. However, the SAPREL technology would allow in future studies a sample for in-depth study to be purposively sampled and therefore to be more representative of the population under study. This approach has the potential to quantify any selection bias and allow researchers to avoid or adjust for it.

Linking data at the individual level across care boundaries offers the opportunity of evaluating system impact of policy initiatives which cannot be effectively measured separately in different sectors of the health and social care provision. The resulting file tracks a cohort of individual patients through the system across primary and secondary healthcare organisational boundaries, and avoids the potential biased conclusion through the analysis of different cohorts of patients from separate cross-sectional databases.

### Comparison with the literature

There are two approaches to achieving record linkage probabilistic (making the most likely matches) and deterministic (requiring precise matching). Probabilistic linking requires the readable sensitive values from different data stores to be brought together for similarity checking. This approach increases the breach risk of that data by creating a larger pool and so reduces the number of sites willing to contribute health data [18,19]. For these reasons SAPREL is based on the deterministic approach but introduces some probabilistic methods to keep error rates down [20].

Analysis in this paper found that compared with the 2001 census, the study practices' populations had more people of working age (20 to 50 for men, and 20 to 35 for women), and a larger proportion of children aged under 5 [21]. Other studies and household surveys conducted in the UK report a higher prevalence of common mental health problems in females [22,23]. Deprivation is associated with poor outcomes including in mental health, so the accessibility of the IAPT service to people of low socioeconomic status in important [24-26].

Strategies such as placing researchers with honorary contracts into practices have been suggested as an alternative method of accessing records [27], methods have also been piloted to use "agents" (software to flag eligible patients) to meet this need [28]. However, all of these methods, including SAPREL rely on the clinicians responsible for patient's health data having trust in the professionalism of the person extracting these data [29].

### Limitations of the method

Our reporting of ethnicity could have been more complete. We could have also increased the ethnicity recording using data from hospital information but did not retain this data field [30].

Recruitment of patients for research projects is challenging; and it is possible that older patients had more time available to participate. The fuzzy logic linkage used in SAPREL was not independently checked for accuracy. We did not find any contradictions in these data, however this does not mean there was a perfect match. We feel that this linkage is sufficiently good for a research study but because there will be errors (false positive and false negative) it is not recommended as a method for identifying the care in practice of individual patients. We only had permission to link these data privately so cannot precisely comment precisely on their performance metrics. However, we presume linkage accuracy close to the 87-88% reported in similar approaches [31]. Both these methods are likely to be progressively improved over time with higher rates of matching achieved [32]. Where sensitive data can be pooled probabilistic methods may ultimately outperform deterministic methods [33].

It is challenging to defend whether private data linkage is truly linking the data we claim it is. We could not go beyond comparing the age and IMD in the linked sample with the unlinked people referred to IAPT. The linked IAPT referred group were a much better (though not perfect) match to the unlinked IAPT referred group than either the groups referred to hospital or in-depth evaluation group. In future studies we will build in the capacity to check the match in demographics more thoroughly.

Data quality is always an issue for studies using routine data [34], and our CMHP category relies on the clinician coding the problem title. Problem titles not always entered into primary care records and the nature of the short consultation in primary care means that not all data are recorded; incompleteness of data is a considerable limitation in its interpretation. Data quality in mental health is challenging [35]. People without a mental health problem coded in their computer record may have had appropriate data recorded as free-text or have a physical health problem label (e.g. headache) [36].

### Call for further research

Further research is needed to assess the acceptability of this approach to patients and to test its reliability - using a dataset where we can do open matching as well as using the SAPREL fuzzy logic.

## Conclusions

Patients referred to the IAPT are predominantly of working age, i.e. aged between 16 and 64 and white population is overrepresented in the IAPT referred group. The sample who volunteered for the in-depth IAPT study was not entirely representative of the total population referred to the IAPT programme. They tended to be drawn from less deprived areas, were even more likely to be female, and older. These biases must be borne in mind when attempting to extrapolate the findings of the study to other populations. Linking data using SAPREL, a flexible largely deterministic method of private data linkage, has been shown to be technically feasible, ethically acceptable and has provided insight into selection bias.

## Conflict of interest

SdeL: None

TC: None

RN: Technical Director of Sapior Ltd - who supply privacy enhanced solutions for the collection, de-identification and linking of sensitive data. The algorithms and fuzzy logic used are the intellectual property of Sapior.

GP: None

KD-B: None

TK: None

## Authors' contributions

All authors read and approved the final manuscript

SdeL: Principal investigator for the data linkage project; overall design and responsibility for the project. Developed the analysis plan with TC, drafted the paper outline and made major contributions to subsequent drafts. Liaised with RN over the detail of the study.

TC: Developed the detailed protocol, managed the ethics and PIAG application, and made major contributions to writing the paper.

RN: Honorary Research Assistant at St. George's and Technical Director of Sapior. Worked with SdeL to explore to how methods designed for private linkage of large datasets could be used in the research context. Used Sapior privacy enhanced fuzzy logic system to link.

GP: PI of NIHR SDO evaluation of IAPT demonstration sites, input into protocol and contributed to the paper.

KD-B: Input into the detailed organisation and running of all stages of the project and contributed to the paper.

TK: Led the write up of the GP data within the NIHR SDO final report and contributed to the paper.

## Pre-publication history

The pre-publication history for this paper can be accessed here:

http://www.biomedcentral.com/1472-6947/11/61/prepub

## Supplementary Material

Additional file 1 **Comparing age and index of multiple deprivation (IMD) in people included in the study with linked practice and IAPT data with those referred to IAPT from non-linked practices**. The frequency of the distribution is shown on the y-axis; IMD data are less complete than age. A normal distribution curve is superimposed showing the left-skew in the age distribution, and right-skew in the distribution of IMD. Click here for file
