# Supplementary figures and images for: Detecting referral and selection bias by the anonymous linkage of practice, hospital and clinic data using Secure and Private Record Linkage (SAPREL): case study from the evaluation of the Improved Access to Psychological Therapy (IAPT) service
Source: BMC Med Inform Decis Mak. 2011 Oct 13;11:61. doi: 10.1186/1472-6947-11-61 (PMC3204226; doi:10.1186/1472-6947-11-61)

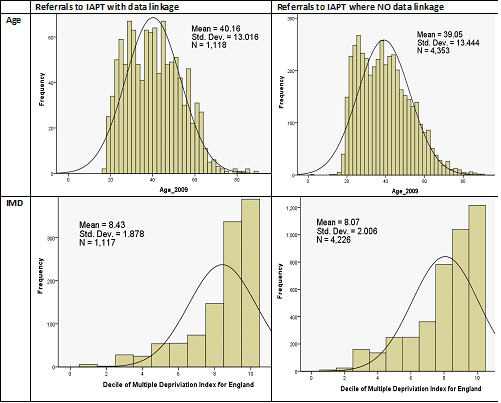

Supplement: Additional file 1 — Comparing age and index of multiple deprivation (IMD) in people included in the study with linked practice and IAPT data with those referred to IAPT from non-linked practices. The frequency of the distribution is shown on the y-axis; IMD data are less complete than age. A normal distribution curve is superimposed showing the left-skew in the age distribution, and right-skew in the distribution of IMD. [file 1472-6947-11-61-S1.TIFF]
